# A novel cell-permeable LOXL2 inhibitor PAT-1251 potently suppresses biliary liver fibrosis via collagen crosslinking-dependent and -independent mechanisms

**DOI:** 10.1097/HC9.0000000000000863

**Published:** 2025-12-12

**Authors:** Ping An, Guangyan Wei, Pinzhu Huang, Heansika Matta, Wenda Li, Yi Lin, Jing Wang, Bain Gretchen, Yury V. Popov

**Affiliations:** 1 Division of Gastroenterology and Hepatology, Renmin Hospital, Wuhan University, Wuhan, Hubei, China; 2 Divison of Gastroenterology and Hepatology, Beth Israel Deaconess Medical Center, Harvard Medical School, Boston, Massachusetts, USA; 3 Department of Radiation Oncology, The First Affiliated Hospital, Sun Yat-sen University, Guangzhou, China; 4 PharmAkea, Inc., San Diego, California, USA

**Keywords:** AB0023, cholangiopathy, cirrhosis, ductular reaction, hepatic progenitor cell, lysyl oxidase-like 2 (LOXL2), pat-1251, primary biliary cholangitis (PBC), primary sclerosing cholangitis (PSC)

## Abstract

**Background:**

LOXL2 promotes fibrosis through extracellular collagen crosslinking and intracellular signaling mechanisms. Here, we studied the mode of action of a novel potent, cell-permeable LOXL2 inhibitor PAT-1251 on hepatic fibrosis.

**Methods:**

PAT-1251 was tested in direct comparison to the anti-LOXL2 mAb AB0023 in the *Mdr2^−/−^* biliary fibrosis model of pre-established fibrosis. The direct cellular effects of PAT-1251 (0.1–10 µM) or AB0023 mAb (30 µg/mL) were studied in primary HSC and EpCAM+ progenitor cell (HPC) cultures in vitro.

**Results:**

Both PAT-1251 and AB0023 were effective at inhibiting collagen crosslinking and reducing portal hypertension and serum transaminase (ALT) levels. Histologically, the placebo-treated group developed severe periportal and perisinusoidal fibrosis with bridging, which was markedly attenuated in PAT-1251–treated mice, with up to 77.7% reduction in hepatic collagen deposition with the high-dose PAT-1251. Treatment with the low dose of PAT-1251 or AB0023 resulted in a moderate improvement in hepatic fibrosis and a modest reduction in collagen deposition. PAT-1251, but not AB0023, significantly reduced ductular proliferation and favored hepatocyte-driven liver regeneration in vivo. In vitro, PAT-1251 promoted colony formation and hepatocyte differentiation in EpCAM+ HPC and dose-dependently inhibited α-SMA expression, cell proliferation, and fibrogenic gene expression in HSC, while the anti-LOXL2 antibody AB0023 had no substantial effect.

**Conclusions:**

While having comparable extracellular effects on collagen crosslinking in vivo, the cell-permeable LOXL2 inhibitor PAT-1251 exerted potent antifibrotic activity in hepatic progenitors and HSC cultures compared with the anti-LOXL2 antibody. PAT-1251 substantially outperformed the anti-LOXL2 antibody in the BALB/c. *Mdr2^−/−^* model of biliary fibrosis, suggesting that intracellular LOXL2 targeting is therapeutically important in addition to its well-characterized extracellular collagen crosslinking activity.

## INTRODUCTION

Liver fibrosis is a dynamic wound-healing process characterized by continuous and progressive deposition of extracellular matrix (ECM) affecting large numbers of patients worldwide.^1^ Apart from liver transplantation, therapeutic options for non-viral chronic liver diseases associated with progressive fibrosis, cirrhosis, and its life-threatening complications are extremely limited or lacking.^2^^,^^3^

Therefore, the development of effective and directly acting anti-fibrotic drugs that can halt or reverse the progression of liver fibrosis is urgently needed.^4^

During fibrosis, HSCs are activated and transdifferentiate into proliferative, contractile, fibrogenic myofibroblasts, which produce and secrete large amounts of ECM proteins, as well as enzymes that stabilize ECM components, including fibrillar type I and III collagens via the process of collagen crosslinking.^5^^,^^6^ Collagen crosslinking is a critical process for fibrotic matrix stabilization and progressive accumulation, and limits fibrosis reversibility. As a result, inhibition of collagen crosslinking is considered a promising therapeutic strategy to treat fibrotic diseases.^7^

Irreversible collagen stabilization in liver fibrosis was proposed to be mediated by tissue transglutaminase (TG2) crosslinking.^7^^,^^8^ However, our studies in TG2-deficient mice found that this ubiquitous crosslinking enzyme is dispensable for both progression and regression of liver fibrosis.^9^ Instead, we directly demonstrated that lysyl oxidase (LOX)^10^ activity is critical for hepatic collagen crosslinking and contributes to the irreversibility of fibrotic scars.^11^ Lysyl oxidase-like 2 (LOXL2), which is a member of the LOX family of enzymes, was subsequently identified as both highly upregulated during fibrogenesis^12^ and a major functional contributor to fibrotic matrix stabilization.^13^ AB0023, a therapeutic anti-LOXL2 targeting monoclonal antibody, was discovered and shown to have promising preclinical efficacy in various models of pre-established biliary fibrosis and non-biliary fibrosis in mice.^14^ Interestingly, the biological role of LOXL2 may not be limited to enzymatic extracellular collagen crosslinking, but may also be important for epithelial cell differentiation.^15^^,^^16^ In the liver, the extracellular collagen crosslinking and intracellular hepatic progenitor cell differentiation activities appear to synergistically drive hepatic fibrogenesis.^13^ This raises the question whether cell-permeability would be desirable and/or required for maximal anti-fibrotic efficacy of LOXL2 inhibitors, particularly in the aftermath of the unsuccessful phase 2 clinical trials of the first anti-LOXL2 antibody simtuzumab.^17^

Given the biological importance of LOX family members in pro-fibrotic pathways, targeting LOXL2 was considered a promising therapeutic strategy to address fibrosis and promote its reversal.^12^ Because antibody-mediated targeting is largely restricted to the extracellular compartment, the important question remains whether intracellular LOXL2 activity must also be engaged for maximal therapeutic effect. PAT-1251 is a potent, selective, cell-permeable, and orally efficacious small molecule inhibitor of LOXL2^15^ that has shown efficacy in preclinical models of lung and kidney fibrosis.^18^^,^^19^ Here, we investigated in detail the preclinical in vitro and in vivo activities of PAT-1251 in a biliary fibrosis model and in non-parenchymal liver cell cultures in direct comparison to antibody-mediated LOXL2 neutralization with AB0023.

## METHODS

### Animal experiments

All animals were housed with a 12-hour light–dark cycle and permitted ad libitum consumption of water and a standard chow diet unless otherwise stated. All animal procedures were approved by the Institutional Animal Care and Use Committee at Beth Israel Deaconess Medical Centre (protocols 010-2015, 003-2021-24).

#### BALB/c.*Mdr2^−/−^* mouse model of biliary liver fibrosis

*Mdr2(abcb4)^−/−^* mice on the fibrosis-susceptible BALB/c background, which spontaneously develop accelerated severe biliary fibrosis, early-onset portal hypertension, and liver cancer, were generated and characterized as reported previously^20^ and bred in animal research facilities at BIDMC. Age-matched males were used in all experiments. Treatment started at 6 weeks of age, when advanced liver fibrosis was already established, and continued for the following 6 weeks.

#### LOXL2 inhibitors

PAT-1251, a potent, cell-permeable, orally available small molecule inhibitor of LOXL2, was synthesized at PharmAkea, Inc.^18^ In the BALB/c.*Mdr2^−/−^* model, PAT-1251 was formulated in 0.5% methylcellulose and administered via once daily oral gavage at doses of 30 or 60 mg/kg starting at 6 weeks of age for the following 6 weeks. Males were used in all studies, and doses were selected based on *C*_max_ drug exposure analysis after short-term treatment in 6-week-old *Mdr2^−/−^*, as detailed in Supplemental Material and Supplemental Figure S1, http://links.lww.com/HC9/C205. A parallel group received the anti-LOXL2 mAb AB0023 administered as an intraperitoneal (i.p.) injection at 30 mg/kg twice weekly as a comparator according to a previously established dosing protocol.^13^ Animals in the placebo control group were orally administered the PAT-1251 vehicle (0.5% methylcellulose).

Additional methods, including isolation and culture of primary murine HSCs, hepatic progenitors, colony-forming and differentiation assay, LDL uptake assay, direct invasive portal pressure measurements, immunohistochemistry, immunofluorescence, qRT-PCR, and primers (Supplemental Table S1, http://links.lww.com/HC9/C205), list of antibodies used (Supplemental Table S2, http://links.lww.com/HC9/C205), can be found in the Supplemental Material, http://links.lww.com/HC9/C205.

### Statistical analyses

Data are expressed as means±SEM, and statistical analyses were performed using Microsoft EXCEL and GraphPad Prism version 5.00 (GraphPad Software, San Diego, CA). Multiple comparisons were performed by one-way ANOVA with the Dunnett post-test. In vitro experiments were performed in triplicate and analyzed using ANOVA or *t* test when appropriate. *p*-values lower than 0.05 were considered significant.

## RESULTS

### Delayed treatment with PAT-1251 potently improved liver injury, portal hypertension, and collagen crosslinking in BALB/c.*Mdr2^−/−^* mouse model

**FIGURE 1:**
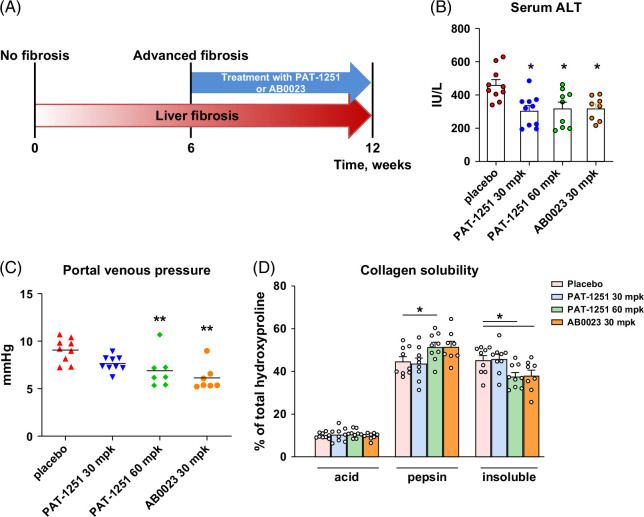
Delayed treatment with cell-permeable LOXL2 inhibitor PAT-1251 and LOXL2-neutralizing mAb AB0023 results in comparable effects on liver injury, portal hypertension, and collagen crosslinking in BALB/c.*Mdr2^−/−^* mouse model. (A) Scheme of delayed treatment with LOXL2 inhibitors. BALB/c.*Mdr2^−/−^* mice with pre-established advanced liver fibrosis were administered with PAT-1251 via daily oral gavage (30 or 60 mg/kg), or anti-LOXL2 mAb (AB0023, 30 mg/kg i/p twice weekly) or vehicle (0.5% methylcellulose) as a placebo control, from 6 to 12 weeks of age (n=9–10). (B) Serum levels of transaminases (ALT) in mice receiving PAT-1251 or AB0023 compared with placebo controls. (C) Portal venous pressure at study endpoint, as determined by direct invasive measurement using a micro-tip pressure monitor. (D) Fibrotic matrix crosslinking was assessed ex vivo using step-wise collagen fractionation as described in the “Methods” section. *p<0.05; **p<0.01 compared with placebo control group (ANOVA with the Dunnett post-test).

PAT-1251 was tested in the male BALB/c.*Mdr2^−/−^* mice, our established model of severe progressive biliary fibrosis. Doses of 30 mg/kg or 60 mg/kg PAT-1251 were experimentally selected based on drug levels achieved in 6-week-old *Mdr2^−/−^* mice, to achieve systemic *C*_max_ drug exposure approximately at IC_50_ and IC_90_ levels (see Supplemental Results and Supplemental Figure S1 for details, http://links.lww.com/HC9/C205). Treatments were administered to mice via daily oral gavage starting from 6 weeks of age, when advanced liver fibrosis is already established. Dosing continued for 6 weeks, after which liver injury, portal pressure, and fibrosis were assessed (Figure 1A). The anti-LOXL2 mAb, AB0023, which previously showed efficacy in BALB/c.*Mdr2^−/−^* mice with early biliary fibrosis stages of this model (4–8 weeks of age), with encouraging results,^13^ was used as a comparator and administered i.p. twice weekly at 30 mg/kg. PAT-1251 was well tolerated at both doses, and no weight loss, death, or other adverse clinical signs were observed (Supplemental Table S3, http://links.lww.com/HC9/C205). Treatment by both PAT-1251 and AB0023 decreased serum ALT levels by ~30% compared with placebo control (*p*<0.05) (Figure 1B). Both PAT-1251 at high dose and AB0023 were equally effective on fibrosis-related portal hypertension, significantly reducing portal venous pressure by an average 2.2 mm Hg and 2.9 mm Hg compared with placebo, respectively (*p*<0.01) (Figure 1C). PAT-1251 at the low dose of 30 mg/kg demonstrated a clear trend to reduced portal hypertension, with average portal venous pressure lowered by 1.7 mm Hg compared with the placebo group, although this did not reach statistical significance. To assess the direct effect of treatments on collagen crosslinking and fibrotic matrix stabilization, a stepwise collagen extraction assay was performed as described previously (Figure 1D).^11^ Both 60 mg/kg PAT-1251 and AB0023 showed similar suppressive effects on the highly crosslinked insoluble collagen fraction, with a one-quarter reduction compared with the placebo control. In both high-dose PAT-1251 and AB0023-treated groups, there was a corresponding increase in pepsin-soluble, moderately crosslinked collagen fraction. Overall, these changes are consistent with robust inhibition of extracellular collagen crosslinking by both treatments.

### PAT-1251 suppresses the progression of biliary fibrosis in BALB/c.*Mdr2^−/−^* mice

**FIGURE 2:**
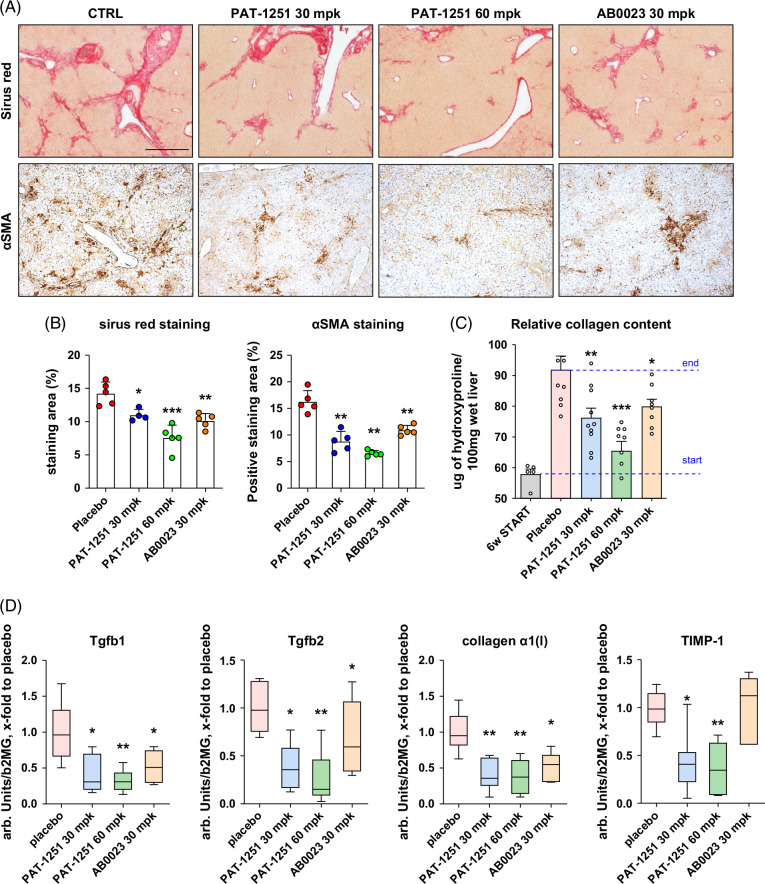
PAT-1251 potently suppresses the progression of hepatic fibrosis in BALB/c.*Mdr2^−/−^* mice, outperforming therapeutic antibody AB2003. (A) Representative images of connective tissue (picrosirius red, upper panel) staining (original magnification, 100×) and immunohistochemistry for α-SMA (lower panel). Representative images shown (original magnification, 200×). (B) Morphometric quantification of collagen area of picrosirius red and αSMA was performed by ImageJ in 10 random portal HPF at 200× magnification (n=5 per group). *p<0.05; **p<0.01 compared with placebo control group (ANOVA with the Dunnett post-test). (C) Hepatic collagen deposition, assessed biochemically via hydroxyproline content (n=5–10). (D) Pro-fibrogenic transcript levels of *Tgfb1*, *Tgfb2*, *Timp1*, procollagen α1(I) (*Col1a1*) as measured by TaqMan qRT-PCR in livers of *Mdr2^−/−^* mice relative to b2MG as housekeeping gene (*x*-fold to healthy control levels). Data are mean±SEM (n=5–10). *p<0.05; **p<0.01 compared with placebo control group (ANOVA with the Dunnett post-test).

Histological analysis of picrosirius red-stained tissue showed that the placebo group developed severe periportal and perisinusoidal fibrosis with bridging, which was markedly attenuated in BALB/c.*Mdr2^−/−^* mice receiving PAT-1251 at both doses (Figure 2A). Morphometric analysis of collagen area showed a dose-dependent reduction in fibrosis with PAT-1251 treatment, with reductions in collagen area of 46.8% and 22.9% in the 60 mg/kg and 30 mg/kg PAT-1251–treated groups, respectively, compared with the placebo control (Figure 2B). While AB0023 also improved fibrosis histologically, the magnitude of the reduction in collagen was substantially lower by morphometric analysis (29% vs. placebo control). Immunohistochemistry for α-SMA, a marker of HSC activation, revealed pronounced accumulation of α-SMA-positive periportal/periductal HSCs in the livers of placebo-treated *Mdr2^−/−^* mice, whereas PAT-1251 markedly and dose-dependently suppressed HSC activation, resulting in a 59.5% (*p*<0.01) and 45.9% (*p*<0.01) reduction in α-SMA-positive area at the 60 mg/kg and 30 mg/kg doses, respectively (Figures 2A, B). Treatment with AB0023 showed a similar effect on α-SMA staining to the low dose of PAT-1251, resulting in a 38% reduction in α-SMA–positive area (Figures 2A, B; *p*<0.01). Biochemically, hepatic collagen levels assessed through hydroxyproline analysis were significantly elevated in the placebo group compared with the 6-week-old BALB/c.*Mdr2^−/−^* “start of treatment” control group (“6W Start” group) (Figure 2C). PAT-1251, dosed at 60 mg/kg, suppressed collagen deposition by 77.7% compared with the placebo control group (*p*<0.05), whereas both the 30 mg/kg dose of PAT-1251 and the AB0023 treatment showed a weaker effect with 46% and 35.1% reductions in hepatic hydroxyproline concentration, respectively. Finally, pro-fibrogenic transcript levels of procollagen α1(I), *Tgfb1*, *Tgfb2*, and *Timp1* were markedly reduced (2–4-fold) in PAT-1251 mice as compared with placebo (Figure 2D). Again, treatment with the LOXL2 mAb AB0023 reduced pro-fibrogenic gene transcript levels to a lesser degree than PAT-1251 treatment.

### Pro-fibrogenic ductular reaction is attenuated by PAT-1251 treatment in BALB/c.*Mdr2^−/−^* mice

**FIGURE 3:**
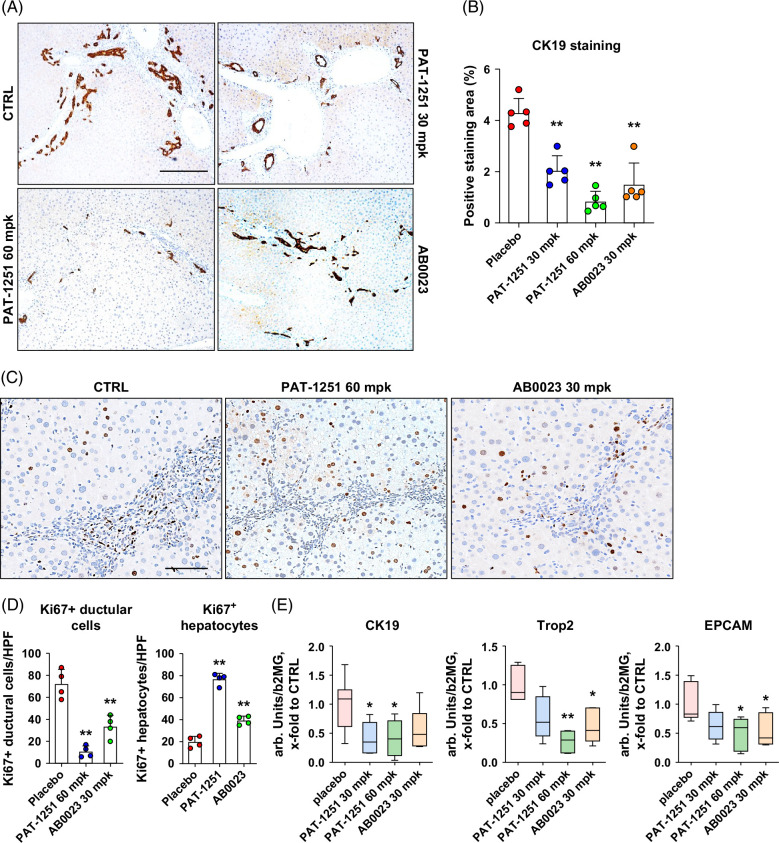
Ductular reaction is markedly attenuated by PAT-1251 treatment in BALB/c.*Mdr2^−/−^* mice, favoring hepatocyte-driven liver regeneration. (A) Representative CK19 immunohistochemistry with (B) morphometric quantification of CK19+ ductal cells (>7 HPF, n=5, 50×) in *Mdr2*−/− mice receiving PAT-1251 or AB0023 compared with placebo controls. (C) Cell proliferation (Ki-67 immunohistochemistry, 200×). Note increased proliferation in ductular structures in placebo controls, whereas replicating hepatocytes predominate in PAT-1251–treated *Mdr2^−/−^* mice. (D) Morphometric quantification of Ki-67+ hepatocytes and ductal cells (>7 HPF, n=4, 200×, one value represented the average of 10 HPF per animal). (E) Gene expression of cholangiocyte-specific (*Krt19*) and hepatic progenitor cell (*Epcam*, *Trop2*) markers as measured by TaqMan qRT-PCR in livers of *Mdr2^−/−^* mice relative to *B2m* as housekeeping gene (*x*-fold to placebo control levels, n=9–15). Data are means ± SEM. *p<0.05; **p<0.01 compared with placebo control group (ANOVA with the Dunnett post-test). All images were acquired and shown in the panel at the same magnification.

We assessed ductular reaction via immunohistochemistry for the ductal cell marker CK19. Ductular reaction is a key pathological process driving biliary liver fibrosis progression by paracrine activation of HSC.^4^ Morphometric analysis of the liver tissue showed that PAT-1251 profoundly and dose-dependently suppressed the expression of CK19 with a 6.1-fold decrease at the 60 mg/kg dose and a 2.1-fold decrease at the 30 mg/kg dose (Figures 3A, B). A significant, but substantially lower, 2.8-fold reduction of CK19-positive area was observed in the livers of AB0023 antibody-treated mice. The PAT-1251–induced suppression of ductular reaction was accompanied by a major pro-regenerative shift in cell replication from mostly ductular cells in the placebo group to predominantly hepatocytes in PAT-1251–treated mice (Figure 3C). Quantitative morphometry revealed a highly significant 6.7-fold reduction in Ki-67(+) ductal cells, paralleled by a 3.8-fold increase in Ki-67(+) hepatocytes in the high dose PAT-1251–treated mice (Figure 3D) and robust suppression of transcripts of ductular reaction/HPC activation markers CK19, Trop2, and EpCAM (Figure 3E). Antibody-mediated LOXL2 neutralization by AB0023 impacted the proliferation of ductular cells and hepatocytes in a similar manner, but to a much lesser degree compared with the 60 mg/kg PAT-1251–treated group (*p*<0.01).

### PAT-1251 promotes the colony-forming ability of hepatic progenitor cells in vitro

**FIGURE 4:**
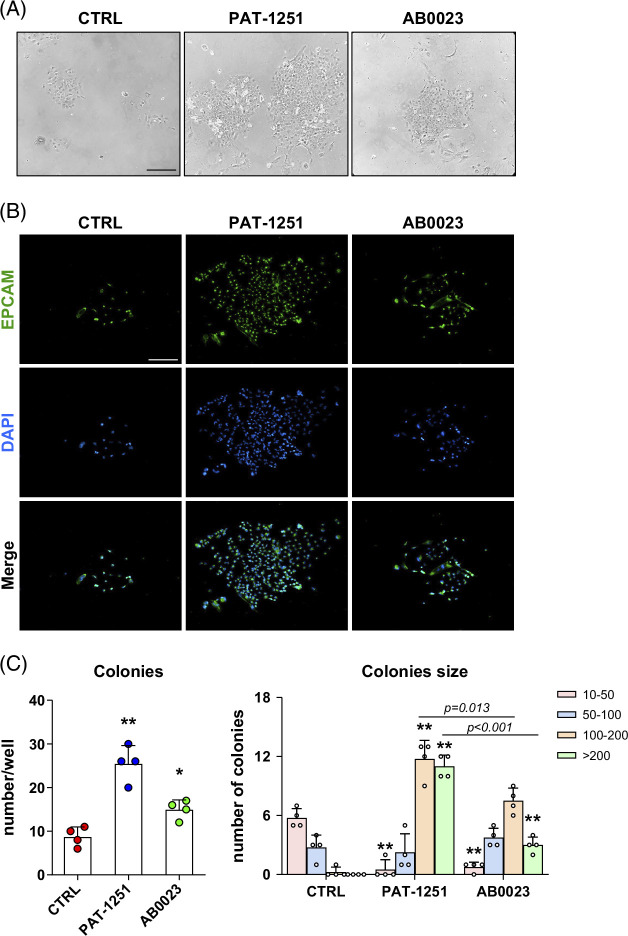
PAT-1251 promotes the colony-forming ability of hepatic progenitor cells in vitro. (A) Primary isolated EpCAM+ hepatic progenitor cells were cultured in differentiation medium for 14 days in the presence of PAT-1251 (10 μM), AB0023 (30 μg/mL), or vehicle, added 12 hours after plating. Representative low magnification (100×) phase-contrast images of cell colonies are shown. (B) EpCAM+ cells (green) in hepatic progenitor cell colonies. PAT-1251 increased the colony-forming ability of EpCAM+ progenitor (oval) cells (original magnification 100×). (C) Colony counts (>50 cells) derived from 1×10^5^ EpCAM+ cultures at day 14 (n=4). *p < 0.05. (D) Colony size categorically graded by cell numbers per colony. Data are means ± SEM. *p<0.05; **p<0.01 compared with vehicle control group (ANOVA with the Dunnett post-test).

Activation of bipotent hepatic progenitor cells is driving “ductular reaction” and is an almost universal finding in chronic liver disease.^21^ To investigate the direct effects of PAT-1251 on HPC biology, we freshly isolated EpCAM+ cells from the chronically injured livers of *Mdr2^−/−^* mice and cultured them in differentiation medium containing 10 μM PAT-1251 or 30 µg/mL AB0023. When cultured in the presence of PAT-1251, HPC-derived cell colonies demonstrated a marked expansion of EpCAM-positive cells (Figures 4A, B). Incubation with the LOXL2-neutralizing mAb exerted a similar, but much weaker effect (Figures 4A, B). Quantification of the colony number and size revealed that PAT-1251 remarkably promoted colony-forming ability of EpCAM+ HPCs, inducing a 2.9-fold increase in overall colony number and 55.5-fold increase in large size colonies defined as >100 cells/colony (Figure 4C). AB0023 treatment showed a weaker, but still significant, effect in HPC colony formation, with 1.9-fold and 14-fold increases in colony number and size, respectively.

### Bipotent HPC differentiation in vitro is redirected from cholangiocyte to hepatocyte lineage in the presence of PAT-1251

**FIGURE 5:**
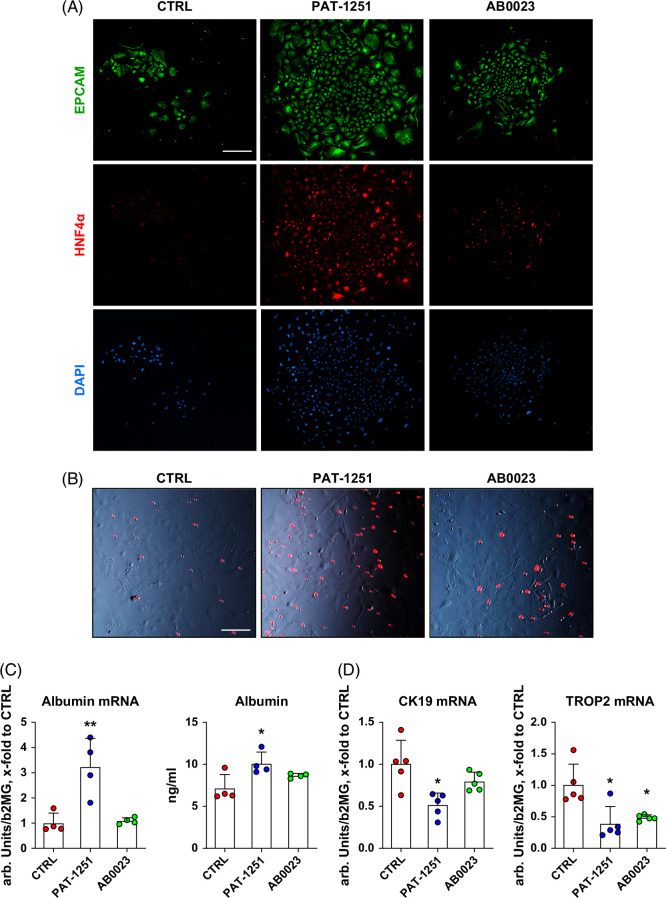
PAT-1251 promotes HPC to differentiate toward the hepatocyte lineage in vitro. (A) Double immunofluorescence for EpCAM (green) and hepatocyte-specific marker (HNF4α, red) in primary EpCAM+ hepatic progenitor cells in the presence of PAT-1251 (10 μM) or AB0023 mAB (30 μg/mL) reveals accumulation of HNF4α-expressing cells in EpCAM(+)-derived colonies. (B) Low-density lipoprotein (LDL) uptake was analyzed using Dil-AcLDL (red fluorescence overlayed on phase-contrast image). (C) Albumin mRNA and protein levels in EpCAM(+) cultures after 14 days of culture with PAT-1251 or AB0023. Data are means ± SEM. *p<0.05; **p<0.01 compared with vehicle control group (ANOVA with the Dunnett post-test). (D) Cholangiocyte (*Krt19*) and progenitor cell (*Trop2*) markers mRNA using RT-PCR were analyzed in total EpCAM+ derived cell colony lysates. Results are representative of 4–5 HPC isolations. Data are means ± SEM. *p<0.05; **p<0.01 compared with vehicle control group (ANOVA with the Dunnett post-test).

Next, we assessed the impact of PAT-1251 on bipotent HPC differentiation, which normally biases toward differentiation to cholangiocytes in vitro.^22^ As determined by in situ immunofluorescence, protein expression of HNF4α, a master hepatocyte differentiation factor, was significantly upregulated in cell colonies from PAT-1251–treated HPC cultures (Figure 5A). In addition, PAT-1251 treatment appeared to remarkably increase LDL uptake, a marker of hepatocyte function (Figure 5B). Further assessment of hepatocyte lineage differentiation was performed by measuring albumin secretion and albumin mRNA expression as bona fide markers of hepatocytes. Based on the albumin endpoints, PAT-1251 treatment appeared to promote the differentiation of HPCs to functional hepatocytes, whereas AB0023 treatment showed only a modest impact (Figure 5C). With respect to cholangiocyte lineage differentiation, treatment with PAT-1251 markedly diminished expression of CK19, a cholangiocyte differentiation marker, in HPC-derived cell colonies (Supplemental Figure S2, http://links.lww.com/HC9/C205). Transcript expression of *Krt19* and *Trop2*, 2 HPC/ductular cell markers, was also significantly downregulated by PAT-1251 treatment, while antibody-mediated LOXL2 neutralization had a lesser effect (Figure 5D).

### PAT-1251 inhibits fibrogenic activation of primary HSCs in vitro

**FIGURE 6:**
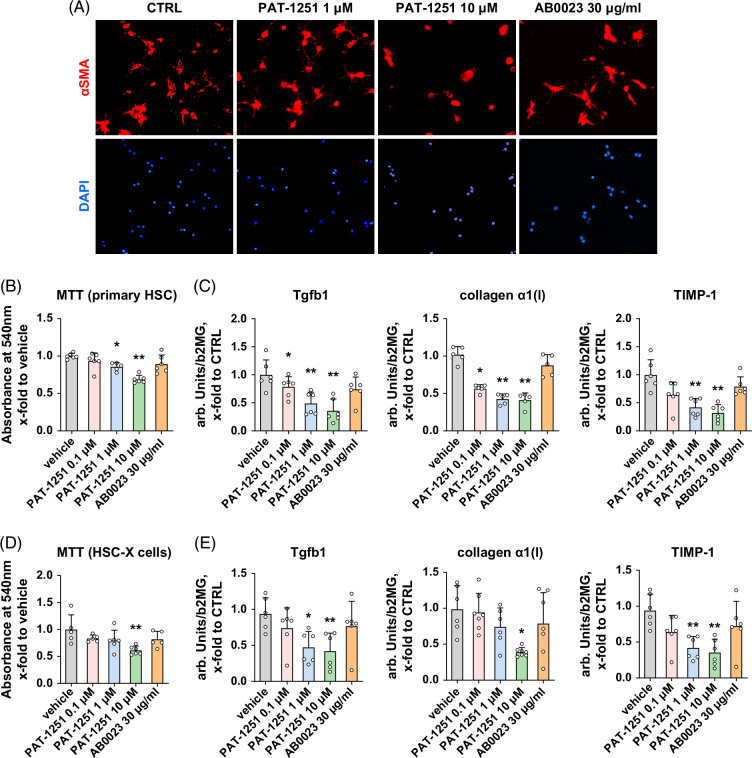
PAT-1251 directly inhibits fibrogenic activation of primary HSCs in vitro. (A) Representative immunofluorescence staining for αSMA (red, nuclei counterstained blue with DAPI, 200×) of primary murine HSC treated with PAT-1251 at 1 and 10 μM, AB0023 at 30 μg/mL, or vehicle. (B) Primary murine HSC treated with PAT-1251 at 0.1–10 μM, AB0023 at 30 μg/mL, or vehicle. Serum-induced cell proliferation of primary murine HSC assessed by MTT assay (n=5–6). Vehicle (DMSO, 0.1%), PAT-1251 at indicated concentrations, or AB0023 mAb added together with 10% FBS. (C) Expression of profibrogenic transcript levels (*Col1a1*, *Tgfb1*, *Timp1*) as quantified by qRT-PCR in primary murine HSC (n=5–6). (D) Serum-induced cell proliferation (MTT assay, n=5–6) and (E). Profibrogenic transcript levels (*Col1a1*, *Tgfb1*, *Timp1*) in rat HSC-X cell line as quantified by qRT-PCR. Results are expressed as mean±SEM, and in arbitrary units (fold to controls) relative to *B2m* mRNA (n=5–6). *p<0.05 and **p<0.01 compared with controls (ANOVA with the Dunnett post-test).

To evaluate the direct effects of PAT-1251 on fibrogenic activation of HSCs, the major effector cells in liver fibrosis, we isolated primary murine HSC from *Mdr2^−/−^* livers and incubated them for 24 hours in vitro with pharmacologically relevant concentrations of PAT-1251 (0.1–10 μM). Expression of the stellate cell activation marker α-SMA was markedly reduced by PAT-1251 at concentrations ≥1 μM, as assessed via immunofluorescence (Figure 6A). Furthermore, PAT-1251 concentration-dependently inhibited serum-induced primary stellate cell proliferation as assessed by the MTT assay and suppressed pro-fibrogenic gene expression of procollagen α1(I), *Tgfb1*, and *Timp1* up to 2–3-fold compared with vehicle controls (Figures 6B, C). Similar results were obtained in the rat activated stellate cell line HSC-X (Figures 6D, E). Treatment with the anti-LOXL2 mAb resulted in a moderate effect on HSC proliferation and activation. Neither PAT-1251 nor AB0023 treatment resulted in any appreciable effect on cell viability as assessed by the trypan blue exclusion test (data not shown).

## DISCUSSION

Recent studies using a neutralizing antibody to LOX2 have revealed that this enzyme plays a critical role in collagen crosslinking and contributes to fibrotic matrix stabilization during hepatic fibrosis progression.^12^ Studies from our group using the LOXL2 targeting antibody have also demonstrated a highly relevant, but previously unknown, role of LOXL2 in driving HPC differentiation toward the ductular lineage, at the expense of the hepatocyte lineage.^13^ Clinical trials have been conducted to address the role of LOXL2 in fibrosis using a humanized therapeutic antibody. The results of these clinical trials were disappointing despite promising pre-clinical data.^13^^,^^14^^,^^23^ Thus, the question arose whether the LOXL2 within the scar tissue was sufficiently engaged by the neutralizing antibody, and/or whether the intracellular activity of LOXL2 must also be inhibited for a robust therapeutic effect.^12^ Here, we show that the novel cell-permeable and selective LOXL2 inhibitor, PAT-1251, demonstrated substantially improved efficacy in vivo and in vitro compared with the LOXL2 targeting antibody, thus suggesting that intracellular LOXL2 activity is an important therapeutic target along with its well-characterized extracellular matrix crosslinking function.

Selective LOXL2 inhibition with the blocking antibody AB0023 efficiently suppressed advanced fibrotic responses in both BALB/c.*Mdr2^−/−^* and DDC (3,5-diethoxycarbonyl-1,4-dihydrocollidine)-induced models of biliary fibrosis in mice.^13^ As the first LOX family member to be specifically targeted, the anti-LOXL2 mAb simtuzumab was broadly evaluated in phase 2 clinical studies for patients with myelofibrosis,^24^ idiopathic pulmonary fibrosis (IPF),^25^ HCV/HIV-induced advanced liver fibrosis,^26^ NASH-associated bridging fibrosis/compensated cirrhosis,^17^ and primary sclerosing cholangitis (PSC).^27^ These studies reported a disappointing lack of effects on tissue fibrosis. Inherent antibody properties, and those specific to AB0023/simtuzumab molecule—such as (partial) allosteric LOXL2 inhibition,^14^ lack of cell-permeability, and limited penetration into mature scar tissue may have contributed to the lack of clinical efficacy as proposed by us^12^ and demonstrated experimentally by others.^28^ AB0023 is a relatively poor LOXL2 inhibitor, achieving only 50% inhibition of enzymatic activity in vitro,^14^ which may be another potential explanation for the disappointing clinical trial results. Therefore, we hypothesized that a potent, cell-permeable small molecule inhibitor of LOXL2 may effectively engage pathological intracellular LOXL2 activities and overcome these pitfalls.

In this study, we evaluated the therapeutic efficacy of the potent, selective, and cell-permeable LOXL2 inhibitor, PAT-1251, in direct comparison with the anti-LOXL2 mAb AB0023 using a relevant in vivo model of progressive biliary fibrosis (BALB/c.*Mdr2^−/−^*) and in vitro systems, including primary hepatic progenitor and HSC cultures. In vivo, we conducted comprehensive efficacy testing of PAT-1251 in the preclinical BALB/c.*Mdr2^−/−^* murine model of chronic liver disease associated with progressive biliary fibrosis.^11^ Delayed therapeutic treatment with PAT-1251 or AB0023 improved liver injury to a comparable extent, as evidenced by the reduction in serum ALT concentration (Figure 1B). AB0023 and the high dose of PAT-1251 also showed similar effects in reducing portal venous pressure, a reliable surrogate predictor of progression to cirrhosis in humans (Figure 1C). Interestingly, both the small molecule inhibitor and the antibody treatment effectively reduced mature and moderately crosslinked collagens, with an identical magnitude of effect, thus strongly suggesting that intracellular LOXL2 is dispensable for the extracellular matrix crosslinking function of LOXL2 (Figure 1D).

Further investigation in vivo revealed several important fibrosis-related parameters where PAT-1251 markedly outperformed the AB0023 antibody. While the anti-LOXL2 mAb had a modest impact on collagen deposition, PAT-1251 robustly and dose-dependently reduced liver fibrosis as assessed by both histological analysis of collagen deposition (Figures 2A, B) and biochemical analysis of hepatic hydroxyproline content. This was accompanied by a profound and dose-dependent effect of PAT-1251 on activation of periportal/periductal HSC, the major fibrogenic effector cell,^3^ with a ~59% reduction in α-SMA–positive area at the high dose of PAT-1251, compared with the more modest ~37% reduction by the anti-LOXL2 antibody (Figure 2A). Likewise, hepatic mRNA expression of several key profibrogenic factors was potently decreased 2–4-fold by PAT-1251, compared with about half of that effect in the antibody-treated group (Figure 2D). Dose-dependent impact was not observed in the reduction of ALT levels (Figure 1B, which was unexpected and likely indirect due to improvement in fibrosis and liver function) and dynamic fibrogenic activity parameters such as mRNA (Figure 2D). Interestingly, PAT-1251's effect on fibrosis (measured by connective tissue area, hepatic hydroxyproline, and α-SMA expression; Figure 2) was apparent already at low dose (30 mg/kg), whereas only high dose suppressed collagen crosslinking (Figure 1D). This may suggest that intracellular (collagen crosslinking independent) LOXL2 activity inhibition manifests efficacy at lower doses and further underscores its relative importance in driving fibrosis.

We acknowledge several limitations of our study. Although our in vitro data unequivocally show that LOXL2 impacts HPC differentiation, our study lacks direct demonstration of lineage differentiation through genetic lineage tracing experiments. Further, the molecular machinery mediating intracellular effects of the LOXL2 inhibitor in HPC and HSC is not yet elucidated. Finally, our efficacy data are limited to a single BALB/c.*Mdr2^−/−^* model of cholangiopathy, which nonetheless has many advantages over existing chronic biliary disease models.^20^ It appears interesting to also explore LOXL2 efficacy in non-biliary, post-necrotic liver fibrosis models not associated with profound ductular reaction. These important questions should be addressed in future studies.

Importantly, PAT-1251 exerted a distinct impact on in vivo liver regeneration. Using in situ hybridization, we have previously pinpointed hepatic LOXL2 expression in the liver to hepatic progenitor cells (HPCs), which are capable of differentiation into both hepatocytes and cholangiocytes.^6^ Differentiation of liver epithelium in vivo was largely redirected from “pathological” HPC-driven regeneration characteristic of fibrotic, chronically injured liver, toward “normal” hepatocyte-driven liver regeneration observed in non-fibrotic, healthy settings, as demonstrated through morphometry of Ki-67+ epithelial cells.^29^ The anti-LOXL2 antibody demonstrated a similar, but substantially less profound, effect in reducing KI-67+ ductular cell counts and promoting hepatocyte replication as compared with the high dose of PAT-1251 (Figure 3). Based on both prior reports and our data, such an effect on this critical physiological process in chronic liver disease is most likely attributed to the intracellular action of LOXL2.^13^^,^^15^^,^^16^ The cell-permeability of PAT-1251 allows for more efficient inhibition of the intracellular pool of LOXL2. These observations are consistent with a previously well-recognized profibrogenic role of ductular reaction, which is characterized by the proliferation of adult bipotent hepatic progenitors and their progeny, including reactive cholangiocytes and intermediate hepatocytes. Ductular reaction is a universal finding in chronic liver disease, but it is particularly profound in biliary disorders such as primary sclerosing cholangitis (PSC), primary biliary cholangitis (PBC), and biliary atresia (BA).^21^ Thus, our data provide a mechanistic basis for the potent, direct therapeutic effect of PAT-1251 on liver injury and hepatic fibrosis compared with AB0023, which acts primarily in the extracellular compartment. The additional effects of PAT-1251 treatment were accompanied by decreased HSC activation (Figure 2A) and downregulation of profibrogenic genes, including Collagen α1, TIMP-1, and transforming growth factor β1 and β2 (Figure 2D), suggesting that the profibrogenic ductular reaction is effectively suppressed by PAT-1251 in vivo, resulting in favorable, hepatocyte-driven regeneration (Figure 3D). It is likely that irreversible nature^18^ of LOXL2 inhibition by PAT-1251 also contributes to its improved efficacy compared with AB023 mAb; recent report of another LOXL2 inhibitor SNT-5382 directly demonstrated a critical role of irreversible substrate binding over its reversible analogue obtained via its chemical modification.^30^ Taken together, these data suggest additional efficacy with combined and irreversible inhibition of both extracellular and intracellular LOXL2.

We further investigated the presumed direct effect of intracellular LOXL2 inhibition by PAT-1251 on HPC differentiation in vivo by using primary cultures of HPC in vitro for in-depth investigation of colony-forming ability and lineage differentiation using established in vitro assays.^13^^,^^22^^,^^31^ We were able to demonstrate a direct stimulation of colony-forming function (Figure 4), as well as pro-repair differentiation toward hepatocyte lineage upon LOXL2 inhibition with PAT-1251 at therapeutically relevant concentrations. Again, while the AB0023 antibody also demonstrated such activity in EpCAM+ HPC cultures, as previously shown by us,^13^ the magnitude of the changes was modest in comparison to PAT-1251 (Figures 4–6). Taken together, these data suggest that treatment with the small molecule LOXL2 inhibitor PAT-1251 exerted a remarkable effect on HPC lineage differentiation that was associated with resultant suppression of ductular proliferation in vivo, underlying its significant therapeutic effect on fibrotic responses in chronic biliary disease.

HSCs and myofibroblasts, the central ECM-producing cells in liver fibrosis, are themselves another major cellular source of most LOX family members in liver fibrogenesis, including LOXL2.^6^^,^^32^ Recent studies have shown that HSC-derived LOXL2 is an important paracrine factor for HPC activation and LOXL2-mediated HPC–HSC crosstalk.^13^ LOX and LOXL inhibition by the non-selective inhibitor BAPN decreased liver stiffness and liver HSC/myofibroblast activation, paralleled by a reduction in the number of α-SMA positive cells in rodent models of liver fibrosis.^11^^,^^32^ However, in our prior studies with antibody neutralization of LOXL2, we observed only indirect effects when HSC were exposed to HPC-derived conditioned medium. No significant direct impact on fibrogenic HSC status could be detected (not shown).^13^ This prompted us to investigate whether the cell-permeable small molecule LOXL2 inhibitor exerts direct effects on isolated HSC activation in vitro. Our results in both primary murine HSCs and the rat HSC-X cell line (Figure 6) clearly show the direct, concentration-dependent inhibition of fibrogenic activity by small molecule LOXL2 inhibition, whereas the anti-LOXL2 mAb failed to substantially impact key HSC activities, including proliferation and fibrogenic gene expression, most likely due to the inability to engage intracellular LOXL2 (Figure 6). Taken together, our data clearly suggest the importance of intracellular LOXL2 function in driving disease-relevant pathology in non-parenchymal liver cells. In addition to blocking well-characterized collagen crosslinking activity in the extracellular space, new generation LOXL2 inhibitors should aim to engage intracellular LOXL2 to achieve a meaningful therapeutic effect in chronic liver disease.

In conclusion, here we report the comprehensive analysis of the therapeutic efficacy of PAT-1251, a cell-permeable selective inhibitor of LOXL2, in a clinically relevant model of biliary liver fibrosis. PAT-1251 substantially outperforms the anti-LOXL2 antibody, especially in regard to anti-fibrotic and pro-regeneration effects. Our data suggest that, in addition to its extracellular collagen crosslinking activities, intracellular LOXL2 plays important pathologic roles in driving fibrogenic response and suppressing hepatocyte-mediated regeneration and thus represents an important pharmacological target. The substantially improved therapeutic efficacy and safety profile of PAT-1251 reported here warrants its further evaluation in clinical trials for biliary and other chronic liver diseases, and informs the rational design of novel small-molecule LOXL2 inhibitors.

## Supplementary Material

## References

[1] Friedman SL Pinzani M . Hepatic fibrosis 2022: Unmet needs and a blueprint for the future. Hepatology. 2022;75:473–488. 34923653 10.1002/hep.32285 PMC12179971

[2] Schuppan D Afdhal NH . Liver cirrhosis. Lancet. 2008;371:838–851. 18328931 10.1016/S0140-6736(08)60383-9 PMC2271178

[3] Kisseleva T Brenner D . Molecular and cellular mechanisms of liver fibrosis and its regression. Nat Rev Gastroenterol Hepatol. 2021;18:151–166. 33128017 10.1038/s41575-020-00372-7

[4] Popov Y Schuppan D . Targeting liver fibrosis: Strategies for development and validation of antifibrotic therapies. Hepatology. 2009;50:1294–1306. 19711424 10.1002/hep.23123

[5] Tsuchida T Friedman SL . Mechanisms of hepatic stellate cell activation. Nat Rev Gastroenterol Hepatol. 2017;14:397–411. 28487545 10.1038/nrgastro.2017.38

[6] Perepelyuk M Terajima M Wang AY Georges PC Janmey PA Yamauchi M . Hepatic stellate cells and portal fibroblasts are the major cellular sources of collagens and lysyl oxidases in normal liver and early after injury. Am J Physiol Gastrointest Liver Physiol. 2013;304:G605–G614. 23328207 10.1152/ajpgi.00222.2012 PMC3602686

[7] Issa R Zhou X Constandinou CM Fallowfield J Millward-Sadler H Gaca M . Spontaneous recovery from micronodular cirrhosis: Evidence for incomplete resolution associated with matrix cross-linking. Gastroenterology. 2004;126:1795–1808. 15188175 10.1053/j.gastro.2004.03.009

[8] Grenard P Bresson-Hadni S El Alaoui S Chevallier M Vuitton DA Ricard-Blum S . Transglutaminase-mediated cross-linking is involved in the stabilization of extracellular matrix in human liver fibrosis. J Hepatol. 2001;35:367–375. 11592598 10.1016/s0168-8278(01)00135-0

[9] Popov Y Sverdlov DY Sharma AK Bhaskar KR Li S Freitag TL . Tissue transglutaminase does not affect fibrotic matrix stability or regression of liver fibrosis in mice. Gastroenterology. 2011;140:1642–1652. 21277850 10.1053/j.gastro.2011.01.040 PMC3374132

[10] Kagan HM Li W . Lysyl oxidase: Properties, specificity, and biological roles inside and outside of the cell. J Cell Biochem. 2003;88:660–672. 12577300 10.1002/jcb.10413

[11] Liu SB Ikenaga N Peng Z Sverdlov DY Greenstein A Smith V . Lysyl oxidase activity contributes to collagen stabilization during liver fibrosis progression and limits spontaneous fibrosis reversal in mice. FASEB J. 2016;30:1599–1609. 26700732 10.1096/fj.14-268425

[12] Chen W Yang A Jia J Popov YV Schuppan D You H . Lysyl oxidase (LOX) family members: Rationale and their potential as therapeutic targets for liver fibrosis. Hepatology. 2020;72:729–741. 32176358 10.1002/hep.31236

[13] Ikenaga N Peng ZW Vaid KA Liu SB Yoshida S Sverdlov DY . Selective targeting of lysyl oxidase-like 2 (LOXL2) suppresses hepatic fibrosis progression and accelerates its reversal. Gut. 2017;66:1697–1708. 28073888 10.1136/gutjnl-2016-312473 PMC5561383

[14] Barry-Hamilton V Spangler R Marshall D McCauley S Rodriguez HM Oyasu M . Allosteric inhibition of lysyl oxidase-like-2 impedes the development of a pathologic microenvironment. Nat Med. 2010;16:1009–1017. 20818376 10.1038/nm.2208

[15] Herranz N Dave N Millanes-Romero A Morey L Díaz V Lórenz-Fonfría V . Lysyl oxidase-like 2 deaminates lysine 4 in histone H3. Mol Cell. 2012;46:369–376. 22483618 10.1016/j.molcel.2012.03.002

[16] Lugassy J Zaffryar-Eilot S Soueid S Mordoviz A Smith V Kessler O . The enzymatic activity of lysyl oxidas-like-2 (LOXL2) is not required for LOXL2-induced inhibition of keratinocyte differentiation. J Biol Chem. 2012;287:3541–3549. 22157764 10.1074/jbc.M111.261016 PMC3271007

[17] Harrison SA Abdelmalek MF Caldwell S Shiffman ML Diehl AM Ghalib R . Simtuzumab is ineffective for patients with bridging fibrosis or compensated cirrhosis caused by nonalcoholic steatohepatitis. Gastroenterology. 2018;155:1140–1153. 29990488 10.1053/j.gastro.2018.07.006

[18] Rowbottom MW Bain G Calderon I Lasof T Lonergan D Lai A . Identification of 4-(aminomethyl)-6-(trifluoromethyl)-2-(phenoxy)pyridine derivatives as potent, selective, and orally efficacious inhibitors of the copper-dependent amine oxidase, lysyl oxidase-like 2 (LOXL2). J Med Chem. 2017;60:4403–4423. 28471663 10.1021/acs.jmedchem.7b00345

[19] Cosgrove D Dufek B Meehan DT Delimont D Hartnett M Samuelson G . Lysyl oxidase like-2 contributes to renal fibrosis in Col4α3/Alport mice. Kidney Int. 2018;94:303–314. 29759420 10.1016/j.kint.2018.02.024 PMC7523185

[20] Ikenaga N Liu SB Sverdlov DY Yoshida S Nasser I Ke Q . A new Mdr2(−/−) mouse model of sclerosing cholangitis with rapid fibrosis progression, early-onset portal hypertension, and liver cancer. Am J Pathol. 2015;185:325–334. 25478810 10.1016/j.ajpath.2014.10.013

[21] Gupta V Sehrawat TS Pinzani M Strazzabosco M . Portal fibrosis and the ductular reaction: Pathophysiological role in the progression of liver disease and translational opportunities. Gastroenterology. 2025;168:675–690. 39251168 10.1053/j.gastro.2024.07.044 PMC11885590

[22] Dorrell C Erker L Schug J Kopp JL Canaday PS Fox AJ . Prospective isolation of a bipotential clonogenic liver progenitor cell in adult mice. Genes Dev. 2011;25:1193–1203. 21632826 10.1101/gad.2029411 PMC3110957

[23] Zhao M Wang L Wang M Zhou S Lu Y Cui H . Targeting fibrosis, mechanisms and cilinical trials. Signal Transduct Target Ther. 2022;7:206. 35773269 10.1038/s41392-022-01070-3 PMC9247101

[24] Verstovsek S Savona MR Mesa RA Dong H Maltzman JD Sharma S . A phase 2 study of simtuzumab in patients with primary, post-polycythaemia vera or post-essential thrombocythaemia myelofibrosis. Br J Haematol. 2017;176:939–949. 28220932 10.1111/bjh.14501

[25] Raghu G Brown KK Collard HR Cottin V Gibson KF Kaner RJ . Efficacy of simtuzumab versus placebo in patients with idiopathic pulmonary fibrosis: A randomised, double-blind, controlled, phase 2 trial. Lancet Respir Med. 2017;5:22–32. 27939076 10.1016/S2213-2600(16)30421-0

[26] Meissner EG McLaughlin M Matthews L Gharib AM Wood BJ Levy E . Simtuzumab treatment of advanced liver fibrosis in HIV and HCV-infected adults: Results of a 6-month open-label safety trial. Liver Int. 2016;36:1783–1792. 27232579 10.1111/liv.13177 PMC5116256

[27] Muir AJ Levy C Janssen H Montano-Loza AJ Shiffman ML Caldwell S . Simtuzumab for primary sclerosing cholangitis: Phase 2 study results with insights on the natural history of the disease. Hepatology. 2019;69:684–698. 30153359 10.1002/hep.30237

[28] Findlay A Turner C Schilter H Deodhar M Zhou W Perryman L . An activity-based bioprobe differentiates a novel small molecule inhibitor from a LOXL2 antibody and provides renewed promise for anti-fibrotic therapeutic strategies. Clin Transl Med. 2021;11:e572. 34841699 10.1002/ctm2.572 PMC8571951

[29] Kuramitsu K Sverdlov DY Liu SB Csizmadia E Burkly L Schuppan D . Failure of fibrotic liver regeneration in mice is linked to a severe fibrogenic response driven by hepatic progenitor cell activation. Am J Pathol. 2013;183:182–194. 23680654 10.1016/j.ajpath.2013.03.018 PMC3702745

[30] Perryman L Findlay A Baskar J Charlton B Foot J Hamilton R . The small molecule LOXL2 inhibitor SNT-5382 reduces cardiac fibrosis and achieves strong clinical target engagement. Sci Rep. 2025;15:22653. 40596021 10.1038/s41598-025-06312-2 PMC12217349

[31] Peng ZW Ikenaga N Liu SB Sverdlov DY Vaid KA Dixit R . Integrin αvβ6 critically regulates hepatic progenitor cell function and promotes ductular reaction, fibrosis, and tumorigenesis. Hepatology. 2016;63:217–232. 26448099 10.1002/hep.28274 PMC5312042

[32] Georges PC Hui JJ Gombos Z McCormick ME Wang AY Uemura M . Increased stiffness of the rat liver precedes matrix deposition: Implications for fibrosis. Am J Physiol Gastrointest Liver Physiol. 2007;293:G1147–G1154. 17932231 10.1152/ajpgi.00032.2007

