# Supplementary material for: A novel cell-permeable LOXL2 inhibitor PAT-1251 potently suppresses biliary liver fibrosis via collagen crosslinking-dependent and -independent mechanisms
Source: Hepatol Commun. 2025 Dec 12;10(1):e0863. doi: 10.1097/HC9.0000000000000863 (PMC12705046; doi:10.1097/HC9.0000000000000863)
Supplement: Supplementary file 1 [file hc9-10-e0863-s001.docx]

**Supplementary materials**

For a research article “*A novel cell-permeable LOXL2 inhibitor PAT-1251 suppresses biliary fibrosis via collagen crosslinking-dependent and -independent mechanisms*” by Ping An et al.

***Supplementary materials and methods:***

***Isolation and culture of primary murine liver cells and cell lines***

*Partially activated primary murine hepatic stellate cells (HSC)* were isolated from fibrotic Mdr2-/- mice and cultured on plastic as described previously (*1*) with modifications(*2*). Briefly, after *in situ* perfusion of the liver with a 0.5mM EGTA/GBSS solution followed by pronase (0.4 mg/ml, Roche, Indianapolis, IN) and collagenase H (0.09 U/ml, Conc, Roche), a non-parenchymal cell fraction was centrifuged in 10% Nycodenz (Sigma) layered with GBSS, and HSCs were collected from the gradient interface. The isolation and culture of spontaneously immortalized rat HSC cell line HSC-X was described previously(*3*).

*Primary murine hepatic progenitor cells (HPC)* were isolated from a nonparenchymal cell fraction of *Mdr2-/-* mice using commercial EpCAM mAb-conjugated magnetic beads on an AutoMACS Pro Separator (Miltenyi) as described(*4*). A colony-formation and HPC- differentiation assay was performed according to Dorrell et al.(*5*), with modifications as previously described in detail(*3, 4*). Briefly, freshly isolated EpCAM+ cells from *Mdr2-/-* mice were seeded at a density of 10^3-4^ cells/cm^2^ on collagen-coated 12-well or 24-well plastic plates (BD Falcon) or a Nunc Lab-Tek II Chamber Slide System (Cole Parmer). Cell medium (10% fetal bovine serum, Dulbecco’s modified Eagle’s medium) was supplemented with murine epidermal growth factor, murine hepatocyte growth factor (both at 10 ng/mL; Peprotech, Inc.), insulin-transferrin-selenium-ethanolamine (Life Technologies Corp.), and dexamethasone (10^-7^ mol/L; Sigma) and changed every 3 days. Colonies were harvested on day 14, and cell supernatant was collected for albumin determination (mouse albumin enzyme-linked immunosorbent assay kit; Assaypro, St. Charles, MO). Colonies, derived from 10^5^ isolated cells/well and defined as organized cell clusters of at least 20 cells, were counted on day 14.

Cell viability was verified by phase-contrast microscopy as well as the ability to exclude trypan blue. The viability and purity of all cell cultures used for the studies was >95% and >90%, respectively.

***Immunohistochemistry, immunofluorescence and immunoblotting.*** Connective tissue stain (Sirius Red) and immunohistochemistry/immunofluorescence were performed in formalin-fixed paraffin-embedded liver sections or snap-frozen liver pieces, as described previously(*4*). Morphometric quantification of collagen area (picrosirius red), immune-positive area for αSMA and CK19 staining in BALBc.Mdr2-/- mice were performed using ImageJ software (NIH, Bethesda) in >10 random high-power fields (HPF) at 200x magnification in at least four individual mice/group. To quantify ductular reaction in PAT-1251 and AB0023-treated mice, morphometric analysis was performed by counting positive cells in >10 random portal tracts of at least four individual mice/group as described(*3*). Detailed information about primary antibodies used is summarized in **Suppl. Table 1**.

***Quantitative reverse transcription polymerase chain reaction (qRT-PCR)***

250-300mg liver tissue from two lobes was homogenized and total RNA was extracted using RNeasy Plus Mini Kit (Qiagen), and 1µg of total RNA reverse transcribed. Relative transcript levels were quantified by real-time RT-PCR on a LightCycler 1.5 instrument (Roche, Mannheim, Germany) using the TaqMan methodology as described previously. TaqMan probes (dual-labeled with 5’-FAM and 3’-TAMRA) and primers (**Suppl. Tab. 2**) were designed using the Primer Express software (Perkin Elmer, Wellesley, USA), synthesized at Eurofins (Louisville, KY), and validated as described(*6*). The housekeeping gene beta-2 microglobulin (β2MG) was amplified in parallel reactions for normalization.

***Hepatic hydroxyproline determination***

Hepatic collagen content was determined as relative hydroxyproline (µg/g liver) in 250-300 mg liver samples from two different lobes (representing >10% of whole liver) after hydrolysis in 6N HCl for 16h at 110^O^C as described(*7*). Total hydroxyproline (mg/whole liver) was calculated based on individual liver weights and the corresponding relative hydroxyproline content(*7, 8*).

***Fibrotic matrix stability assessment***

Fibrotic matrix stability was assessed biochemically ex vivo by complete collagen fractionation through serial extractions(*9*). Five hundred mg of snap-frozen tissue from two liver lobes was homogenized and a series of overnight extractions (1:20, w:v) under increasingly harsh conditions were performed to obtain the following collagen-containing fractions: acetic acid-soluble (non-crosslinked collagens and pro-collagens); pepsin-soluble (fibrillar, mature and moderately cross-linked collagens); and insoluble (the remaining highly cross-linked collagens). Collagen content in each fraction was quantified via hydroxyproline determination after complete acidic hydrolysis and expressed as percentage of hydroxyproline recovered in all fractions.

***Serum biochemistry***

Serum levels of alanine aminotransferase (ALT) were measured using Catalyst Dx^®^ Chemistry Analyzer (IDEXX Laboratories, Inc, Westbrook, ME) according to manufacturer’s recommendations.

***MTT proliferation assay***

Cell proliferation was measured by a MTT [3-(4,5-dimethylthiazol-2-yl)- 2,5-diphenyltetrazoliumbromide] assay (ATCC). Cells were plated at a density of 5x10^3^ cells per well in 96-well culture plates. After treatment, MTT solution was added to the culture medium (0.5 mmol/L) and incubated for 2 hours at 37°C with 5% CO_2_. Detergent solution was then added to solubilize formazan crystals. Optical density was determined at 540 nm using a Benchmark Plus microplate reader (Bio-Rad, Hercules, USA).

***Low-density lipoprotein (LDL) uptake assay***

An LDL uptake assay (ab133127, Abcam, Cambridge, USA) was performed according to the manufacturer's recommendations. Briefly, at the end of described treatments we replaced culture medium from HPCs for the medium containing LDL-Dylight 550 solution. After incubation for 4 hours, we replaced LDL-DyLight550 solution with fresh culture medium and analyzed LDL uptake under fluorescent microscope.

***Direct invasive PVP measurements***

Portal venous pressure (PVP) was measured at the study endpoint as previously established (*6*). Briefly, 1.2-Fr high-fidelity pressure catheter (Scisense, London, ON, Canada) was inserted into portal vein of an anaesthetized mouse after midline abdominal incision. Pressure signals were recorded at 2 kHz for 5 min, and analyzed using PowerLab software chart 5.5.6 (ADInstruments, Colorado Springs, CO, USA), followed by cardiac puncture/exsanguination and tissue collection for analysis.

***Supplementary Results:***

**PAT-1251 dose selection based on plasma and liver drug exposure in Mdr2-/- mice**

A key consideration for advancing molecules into in vivo efficacy studies is ensuring that appropriate drug concentrations are achieved in the tissue(s) of interest. This is of particular importance in pre-clinical studies using mouse models of chronic liver disease such as BALB/c.Mdr2-/-, which may have altered drug metabolism compared to data obtained in formal PK studies in healthy mice due to liver function impairment. PAT-1251 is an irreversible inhibitor with mouse whole blood IC_50_ and IC_90_ values of 0.86 and 5.6 µM, respectively (*10*). A major advantage of irreversible inhibition is the potential for an extended pharmacodynamic response that outlasts systemic pharmacokinetics. Based on this data, our expectation is that if we inhibit LOXL2 at >90% at C_max_, this degree of inhibition would last for at least 24h or at least until significant new LOXL2 protein is synthesized.

To evaluate steady-state PAT-1251 maximum plasma and liver concentrations in the relevant disease model, 6-week-old *Mdr2*-/- mice (corresponding to the starting point of subsequent efficacy studies) were dosed orally, once daily for 3 days with 10 or 30 mg/kg PAT-1251 formulated in 0.5% methylcellulose (n=4-5/group). At 2 hours post last dose, blood and liver tissue were collected and drug concentrations analyzed by LC-MS/MS at Pharmakea, Inc facilities.

**Suppl. Fig. 1** summarizes drug exposure data used to justify the dosage of PAT-1251 in formal efficacy studies. At 2h post-dose, the mean plasma concentrations of PAT-1251 in *Mdr2*-/- mice were 0.124 and 0.347 µM after dosing at 10 and 30 mg/kg, respectively. Liver concentrations were significantly higher than the plasma concentrations with mean values of 1.37 and 2.9 µM after dosing at 10 and 30 mg/kg, respectively. The mean PAT-1251 concentration after dosing at 30 mg/kg was above the mouse blood IC_50_ value of 0.86 µM, but below the mouse blood IC_90_ value of 5.6 µM. Given the incomplete target coverage at C_max_ using a 30 mg/kg dose, we chose this as our low dose level. Given the relatively dose-dependent increase in C_max_ between 10 and 30 mg/kg, our expectation was that a 60 mg/kg dose would result in an approximate C_max_ of 5.8 µM, which is just above the mouse blood IC_90_ value of 5.6 µM. Therefore, we chose 60 mg/kg as our high dose.

**Steady-state Mean PAT-1251 Concentrations (µM) at 2h Post Dose**

| **Tissue** | **10 mg/kg dose** | **30 mg/kg dose** |
| --- | --- | --- |
| Plasma | 0.124 | 0.347 |
| Liver | 1.37 | 2.9 |

**Suppl. Figure 1. Dose-selection study in Mdr2-/- mice. PAT-1251 Mean Plasma and Liver Concentrations (nM) at 2h Post Dose.**

**Suppl.** **Table 1.** **Primary antibodies used in immunochemistry and immunofluorescence staining**

|  | **Primary antibodies** | **Application/Dilution** |
| --- | --- | --- |
| **αSMA(Abcam, ab5694)** | Monoclonal, rabbit anti-mouse | IHC (1:400), IF (1:100) |
| **CK19 (TROMA-III,Hybridoma Bank, Iowa University)** | Monoclonal, rat anti-mouse | IHC (1:200) |
| **Epcam(Abcam, ab32392)** | Monoclonal, rabbit anti-mouse | IF (1:100) |
| **Ki67(Abcam, ab15580)** | Monoclonal, rabbit anti-mouse | IF (1:100) |

**Suppl.** **Table 2.** **Sequences of probes and primers used in real time-PCR**

|  | **5’-primer** | **3’-primer** | **TaqMan Probe** |
| --- | --- | --- | --- |
| **β2MG** | CTGATACATACGCCTGCAGAGTTAA | ATG AATCTTCAGAGCATCATGAT | GACCGTCTACTGGGATCGAGACATGTG |
| **TGFβ1** | AGAGGTCACCCGCGTGCTAA | TCCCGAATGTCTGACGTATTGA | ACCGCAACAACGCCATCTATGAGAAAACCA |
| **Collagen α1(I)** | TCCGGCTCCTGCTCCTCTTA | GTATGCAGCTGACTTCAGGGATGT | TTCTTGGCCATGCGTCAGGAGGG |
| **TIMP-1** | TCCTCTTGTTGCTATCACTGATTAGCTT | CGCTGGTATAAGGTGGTCTCGTT | TTCTGCAACTCGGACCTGGTCATAAGG |
| **Trop2** | TaqMan assay ID: Mm00498401_s1 (Life Technologies) | | |
| **Epcam** | TaqMan assay ID: Mm00493214_m1 (Life Technologies) | | |
| **CK19** | TaqMan assay ID: Mm00492980_m1 (Life Technologies) | | |
| **ALB** | TaqMan assay ID: Mm01354325_m1 (Life Technologies) | | |

**Suppl. Table 3.** Changes in body weight (BW), liver weight, collagen content and spleen weight in Mdr2-/- mice treated with PAT-1251 or AB0023. Body weights, liver weights and spleen weights were measured at sacrifice, and collagen deposition was determined biochemically as relative (μg/g) hydroxyproline (HYP) content in 250mg liver samples from two different lobes. Total HYP(mg/whole liver) was calculated from individual liver weights and respective relative HYP values. Ctrl: healthy mice; 6w start: Mdr2-/- mice of 6 weeks age; Placebo: Mdr2-/- mice treated with vehicle; PAT-1251 30mpk: Mdr2-/- mice treated with PAT-1251 at dose of 30mpk; PAT-1251 60mpk: Mdr2-/- mice treated with PAT-1251 at dose of 60mpk; AB0023 30mpk: Mdr2-/- mice treated with AB0023 at dose of 30mpk i/p. Data are expressed as means ±SEM. *, p<0.05 compared to vehicle control group (ANOVA).

| **Groups** | **Final BW, g** | **Liver/BW**  **×100, %** | **Hyp, ug/wet liver** | **Spleen/BW**  **×100, %** |
| --- | --- | --- | --- | --- |
| **WT (healthy),** *n=5* | 22.74±0.71 | 5.93±0.45 | 226.0±22.44 | 0.35±0.02 |
| **6w Start,** *n=5* | 18.06±1.55 | 8.97±0.40 | 933.8±122.9 | 0.56±0.06 |
| **Placebo,** *n=10* | 24.53±1.14 | 7.43±0.84 | 1611.9±343.4 | 0.82±0.08 |
| **PAT-1251 30mpk,** *n=10* | 26.68±1.58 | 7.50±0.36 | 1502.0±235.9* | 0.84±0.11 |
| **PAT-1251 60mpk,** *n=9* | 25.57±1.48 | 7.11±0.59 | 1380.8±254.1* | 0.80±0.09 |
| **AB0023 30mpk,** *n=9* | 26.05±1.56 | 8.97±0.40 | 1501.1±284.5* | 0.82±0.08 |

**Suppl. Figure 2. PAT-1251 blocks EPCAM+ progenitor (oval) cells differentiation into cholangiocytes in vitro.** Double-immunofluorescence for EpCAM (green) and cholangiocyte-specific marker CK19 (red) in primary EpCAM+ hepatic progenitor cells in presence of PAT-1251 (10μM) or AB0023 mAB (30μg/ml) revealed down-regulation of cholangiocytes in cultured EPCAM+ progenitor cells in presence of PAT-1251 or AB0023 mAb.

**
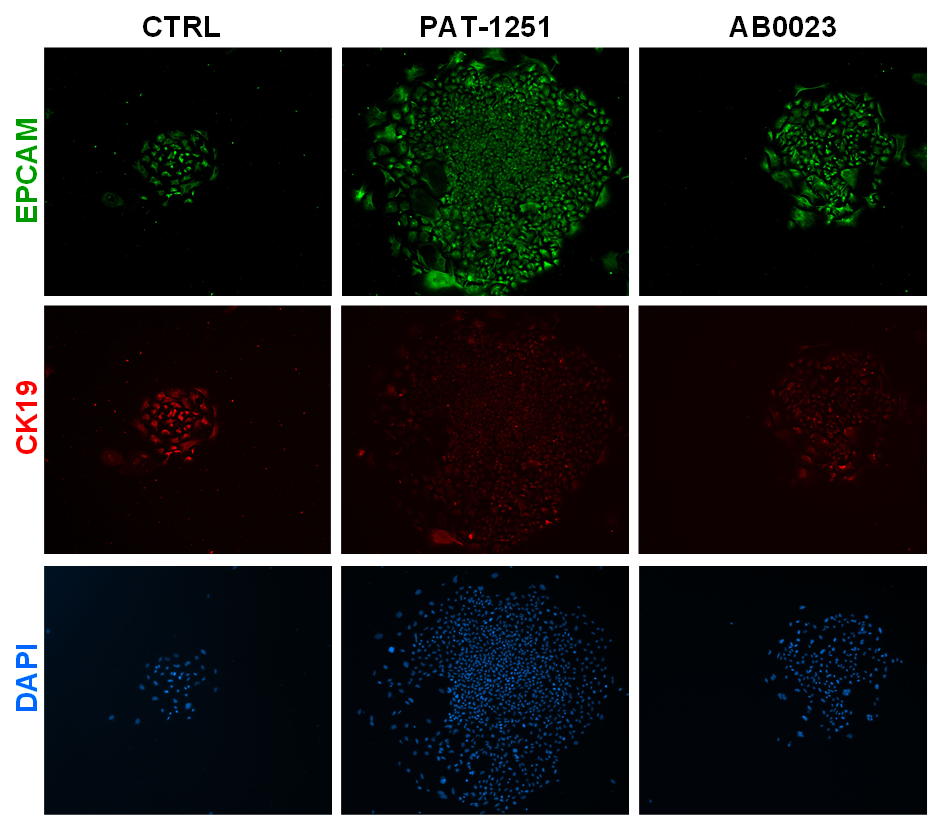
**

***Supplementary references:***

1. I. Mederacke, D. H. Dapito, S. Affo, H. Uchinami, R. F. Schwabe, High-yield and high-purity isolation of hepatic stellate cells from normal and fibrotic mouse livers. *Nat Protoc* **10**, 305-315 (2015).

2. S. Yoshida *et al.*, Extrahepatic Platelet-Derived Growth Factor-beta, Delivered by Platelets, Promotes Activation of Hepatic Stellate Cells and Biliary Fibrosis in Mice. *Gastroenterology* **147**, 1378-1392 (2014).

3. N. Ikenaga *et al.*, Selective targeting of lysyl oxidase-like 2 (LOXL2) suppresses hepatic fibrosis progression and accelerates its reversal. *Gut*, (2017).

4. Z. W. Peng *et al.*, Integrin alphavbeta6 critically regulates hepatic progenitor cell function and promotes ductular reaction, fibrosis, and tumorigenesis. *Hepatology* **63**, 217-232 (2016).

5. C. Dorrell *et al.*, Prospective isolation of a bipotential clonogenic liver progenitor cell in adult mice. *Genes & development* **25**, 1193-1203 (2011).

6. N. Ikenaga *et al.*, A new Mdr2(-/-) mouse model of sclerosing cholangitis with rapid fibrosis progression, early-onset portal hypertension, and liver cancer. *Am J Pathol* **185**, 325-334 (2015).

7. Y. Popov *et al.*, Halofuginone induces matrix metalloproteinases in rat hepatic stellate cells via activation of p38 and NFkappaB. *J Biol Chem* **281**, 15090-15098 (2006).

8. Y. Popov, E. Patsenker, P. Fickert, M. Trauner, D. Schuppan, Mdr2 (Abcb4)-/- mice spontaneously develop severe biliary fibrosis via massive dysregulation of pro- and antifibrogenic genes. *J Hepatol* **43**, 1045-1054 (2005).

9. Y. Popov *et al.*, Tissue transglutaminase does not affect fibrotic matrix stability or regression of liver fibrosis in mice. *Gastroenterology* **140**, 1642-1652 (2011).

10. M. W. Rowbottom *et al.*, Identification of 4-(Aminomethyl)-6-(trifluoromethyl)-2-(phenoxy)pyridine Derivatives as Potent, Selective, and Orally Efficacious Inhibitors of the Copper-Dependent Amine Oxidase, Lysyl Oxidase-Like 2 (LOXL2). *J Med Chem* **60**, 4403-4423 (2017).
